# A Physician’s Perspective on Volunteering Overseas… It Is Not All about Sharing the Latest Technology

**DOI:** 10.3389/fsurg.2017.00077

**Published:** 2018-02-02

**Authors:** Jon Kolkin

**Affiliations:** ^1^Health Volunteers Overseas, Washington, DC, United States

**Keywords:** international, humanitarian, volunteer, medical, missionary, physician, overseas, Health Volunteers Overseas

## Abstract

As health-care professionals, there often comes a time in our career when we are intrigued by the possibility of participating in humanitarian work in underserved countries. However, the desire to serve is often tempered by some ambivalence about whether our skill sets are applicable in less technologically advanced health-care settings. Furthermore, there may be some concern about the cultural and logistical challenges one might face while working overseas. A volunteer may also be worried that, despite their best intentions, the medical personnel in the host country will not take advantage of our knowledge and their patients will not achieve the best results. As a consequence, a talented and well-intentioned professional may decide not to volunteer, resulting in a lost opportunity to participate in what can be an extremely rewarding experience. I will be discussing a number of key factors that can strongly influence the quality of one’s experience from the perspective of a Hand Specialist. My comments will primarily be a reflection of my personal experience over 20 years as a member of Health Volunteers Overseas (www.hvousa.org).

## Introduction

As health-care professionals, there often comes a time in our career when we are intrigued by the possibility of participating in humanitarian work in underserved countries. However, the desire to serve is often tempered by some ambivalence about whether our skill sets are applicable in less technologically advanced health-care settings. This is particularly true of physicians whose decision-making process is heavily dependent on procedures and/or sophisticated equipment. However, just think how refreshing it might be for some of us to be placed in an environment where, out of necessity, we must practice the “art” of medicine, focused primarily on a careful history, the nuances of a detailed physical examination and intuition rather than technology. Also, how liberating not being shackled by electronic medical records!

A prospective humanitarian may be concerned about the cultural and logistical challenges one might face while working overseas. Another potential worry might be that medical personnel in the host country will not take advantage of our knowledge and their patients will not achieve the best results. As a consequence, a talented and well-intentioned professional may decide not to volunteer, resulting in a lost opportunity to participate in what can be an extremely rewarding experience. However, more importantly, if an individual does volunteer but has a “know it all,” “I’m here to teach you the right way” attitude, it can lead to lasting repercussions, doing harm to patients and tempering our hosts’ receptiveness to future efforts by NGOs and their members. In contrast, if one does the proper homework before accepting an assignment and has a healthy mindset, punctuated by humility and a desire to “collaborate” and “exchange” ideas, all parties are more likely to have a rewarding experience. In this article, I will discuss a broad range of key factors that are essential for achieving “success.”

My comments will primarily be a reflection of my personal experience over 20 years as a Hand and Upper Extremity Specialist and member of Health Volunteers Overseas (www.hvousa.org) while working in Vietnam (x3), Myanmar, Moldova (x3), Peru, Nepal, Malawi (x2), Bhutan (x4), India (x2), and China (x2). My perspective is also influenced by a 40-year career as a physician and comments by other healthcare professionals.

## Mindset of an Effective Humanitarian

Humility, compassion, patience, and flexibility are invaluable attributes in a humanitarian. Egos have no place in our work. One must be willing to think creatively, look at a situation from multiple viewpoints and adopt therapeutic strategies to accommodate and respect local conditions, cultures, techniques, politics, resources, educational backgrounds, demographics, and social norms (1).

I believe a healthy mindset can be enhanced by maintaining a critical eye and sensitivity toward using appropriate terminology. As an example, referring to other countries as “third world” subconsciously contaminates the mind, reinforcing an erroneous perception of inferiority. This term can be insulting to our hosts, often interpreted as implying “third rate” status. Consider using more accurate terms such as “underserved” or “under-resourced.” If you come with an open mind and a desire to “collaborate” with respected “colleagues” rather than “teach them how to do things,” you will learn a great deal and your hosts will be more appreciative of you as a human being and receptive to your insights.

## Physician First—Surgeon Second

Physicians who are trained to perform surgery typically assume that their primary activity will be centered around the operating room. This stems, in part, from the conventional practice of referring to ourselves as “surgeons” rather than as “physicians” who are skilled at “helping people” *via* surgical and non-surgical approaches. Emphasizing the benefits of an integrated team approach between physicians, therapists, etc. can further reinforce this positive mindset. Equally important is the physician’s role in educating our patients about factors that may be contributing to their malady along with preventative measures that may lessen their symptoms and the likelihood of surgical intervention. These points are even more crucial in underserved countries where resources are limited and non-surgical approaches may be the most realistic options. Focusing on these crucial roles may also initiate a slight shift in our own mindset, leading us away from being too aggressive surgically, thereby improving the management of patients in our own practice.

## Focus on Fundamentals

Based on my experience, more often than not, the greatest contribution we can make as physicians has very little to do with teaching advanced surgical procedures. Oftentimes, a more valuable contribution involves focusing on fundamental issues that effect a broad segment of the patient population and which can be effectively addressed by dedicating a modest amount of manpower and material resources. As an example, stiff, poorly functioning hands are common in underserved countries. By consistently focusing on maximizing hand function during ward rounds, clinics, informal conversations, lectures, cadaveric dissections, and during surgical procedures, one can highlight the importance of strategic thinking throughout the treatment process.

## The Good Physician—Organization Match

When deciding if and when to serve, it is important to align yourself with an organization whose mission is consistent with your values and objectives. A number of factors may impact your decision such as the population served, out-of-pocket expenses, time commitment, faith, and whether to travel alone or with others. Traveling by yourself may provide advantages such as flexibility in scheduling your trip, greater autonomy, and ease of making adjustments while on site. Traveling with family members can help strengthen personal relationships and provide an opportunity to share your values with the next generation. Traveling with a therapist or other health-care professional can add additional expertise to the experience while also helping to demonstrate the potential benefit of collaborative work. Finally, participating as part of a large group may add an added layer of comfort to first-time volunteers and, in certain situations, provide a level of care unavailable to the local population.

One word of caution: if you decide to align yourself with a religious organization and anticipate “sharing” your religious convictions, I strongly recommend communicating your intentions to your hosts well before either party has invested a significant amount of time and effort into the initiative. If you are not forthright concerning your intentions, even though you may view your actions as honorable, others may view them as deceitful, offering help with “strings attached” and a hidden agenda. Such actions can lead to resentment and mistrust, thereby undermining the goodwill of past and future humanitarians. If your objective is to perform a “Mitzvah,” a Jewish word that means “giving without the expectation of receiving anything in return,” then consider characterizing what you do as “humanitarian work” rather than “missionary work.”

## Assessing Local Medical Conditions

Frequently, I find it helpful to read trip reports and speak with previous volunteers. Representatives of an organization may also be quite knowledgeable.

It is not unusual to walk into a situation where some members of a medical community embrace your presence while others are standoffish, not convinced you will be an asset and/or worried that your presence will threaten their stature within the community. This may be based on their previous experiences, level of confidence in their own skills, and other factors. Alternatively, they may be overwhelmed with patients and mostly interested in another set-of-hands, not a colleague for exchanging ideas. Another dynamic may be their desire to study abroad with aspirations that you will sponsor them and provide financial assistance.

It is important to observe various health-care professionals during clinics, ward rounds, conferences, and the OR in order to assess their temperament, knowledge, and skills. Your observations will be invaluable when assessing whether to suggest a particular method of treatment.

You will also be able to make better recommendations if you understand the dynamics between various departments. As an example, at a teaching institution with 20 hand specialists doing extremely complex microvascular surgery your hosts may have extraordinary technical skill but little understanding of the valuable contributions that can be made by qualified hand therapists. In contrast, you may find yourself with 3 general orthopedists and 20 enthusiastic, engaged and motivated therapists. Clearly, these settings provide different opportunities. After making careful observations, it may be prudent, through tactful conversations, to gauge the level of interest among physicians and therapists to upgrading their relationship. In some instances, you may feel the situation warrants a return visit by yourself or another physician accompanied by a therapist.

It is extremely important to “feel out” the environment for a number of days before diving-in with multiple suggestions. Remember, you are a guest in someone else’s workplace. If you feel your host’s approach seems less than optimum, it is important to recognize that there may be a number of factors unique to their setting, which contribute to their decision-making process. These might include sterility issues in the operating room, wards, and patient’s home; faulty equipment; inaccurate patient records; and spotty availability of certain hospital services. You may not fully appreciate other factors such as limited patient funds, limited OR time, suboptimally trained therapists, and unreliable electricity. Furthermore, your hosts may have had excellent results using other approaches. In the long run, your input will be much more effective if you remain inquisitive. After carefully weighing all factors, it is perfectly reasonable, at the right time and in the right place, to offer your insights and explore various options. A word of caution: your host may strongly disagree with your recommendation but not voice disagreement. This is when you need to be particularly attuned to reading body language, the hesitancy in their voice, the movement of their eyes, and cultural nuances. As an example, they may consider it rude to say “no.” Instead, they may say “yes” with guarded enthusiasm or simply remain silent.

## Learning and Teaching Moments

Whenever possible, find an appropriate time for taking a good history and doing a careful examination rather than relying solely on someone else’s assessment or previous records. You will then be in a better position to give thoughtful insight. By going through this process you will dramatically increase the likelihood of a good outcome and solidify your colleagues’ trust. Using this measured approach also takes advantage of a golden opportunity to demonstrate with actions, not words, the “art” of medicine to the attending physicians, residents, orthopedic assistants, therapists, nurses, and other students.

## When You and Your Host Seem to Disagree

In some instances, the guest’s treatment preferences appear out of synch with those of the host. There may be a number of reasons for this discrepancy. Consider the possibility that your host may have more experience dealing with certain conditions such as chronic osteomyelitis and tuberculosis plus greater familiarity with local factors that might impact results. Alternatively, a host may want to learn how to learn a new procedure in order to advance their skills and/or increase their stature among their colleagues and within their community. Mastering more difficult procedures can also boost their self-confidence and potentially result in greater financial reward. Another possible explanation is that the host may not realize that their health-care system does not have the necessary equipment, trained physical therapies, post-discharge support, etc. needed for achieving optimum results.

A word of caution: always think twice before suggesting the latest-and-greatest, most innovative, and novel approach. You may, at first, be treated with great respect for your unique perspective. However, if things go poorly your credibility may be irreparably tarnished. Furthermore, if you make recommendations that, in the eyes of your hosts, seem too aggressive, radical, or ill advised, you will quickly gain a poor reputation and future input will be suspect.

If after carefully weighing all factors you are still convinced that following your advice would be substantially best for the patient, it is important to find a tactful way of injecting your opinion while graciously acknowledging your host’s efforts and treatment preferences. You might consider saying, “I would really like to hear how you have handled this type of problem in the past”; “Would there be some benefit to considering ‘X’?”; “In my practice I would typically consider doing ‘X’ but do you think taking this approach would be reasonable in your hospital?”; and “I suspect you have more experience with this problem than I do.”

Rarely will you witness a prompt renaissance in thinking or change in practice. More often progress is incremental and often takes place over many years. Therefore, repeat visits can help build on the foundations you and others have initiated.

## Postsurgical Considerations

If surgery is performed it is important not to make assumptions concerning what will happen after you leave the OR. Who will apply the postoperative dressing and splint? Will the intended joints be immobilized and held in the optimum position? Will the splint be applied in the OR, in the recovery room, or on the floor? Do the nurses on the ward have experience in the prevention and monitoring of swelling and the assessment of circulation? When possible take advantage of golden opportunities to demonstrate splint fabrication, circulation assessment, etc. However, please be tactful when demonstrating “proper” technique so as not to offend attending physicians. If the patient is going home after surgery, do not assume anyone has given detailed and/or accurate post-op instructions. Check and double-check!

## Effective Teaching Strategies

More often than not you will be working in a country where English is not the primary language. Even if someone speaks fairly good English, it does not mean they understand all the nuances of a concept you are trying to convey. There are a number of strategies you can use to enhance communication. Consider using short sentences comprised mostly of basic words and limit reliance on medical terminology and acronyms. Speak slowly and calmly while enunciating clearly. This approach will help relax the listener and make it easier for them to concentrate on your words. Hand gestures, scribbling pictures and diagrams on a piece of paper, and demonstrating a technique can also be effective strategies. Laptops, smart phones, and tablets can be useful tools for sharing information. It is prudent to periodically and tactfully probe to see if they understand concepts you are trying to convey. Such opportunities may present themselves in the OR, therapy department, outpatient clinic, nurses’ station, break room, morning report, and impromptu gatherings for meals.

## Additional Landmines to Avoid

Even if you think you clearly articulated a strategy for treating a patient, there are likely to be a number of unforeseen circumstances that impact your effectiveness in achieving optimum results. As an example, family members are often expected to stay at the hospital in order to help care for the patient. However, if no family member is available, the patient may not arrive or may leave prematurely. Also, follow through of instructions may be suboptimum due to: incomplete or abbreviated translation of your instructions; the interpreter not being fluent in the patient’s dialect; cultural attitudes toward personal responsibility; a poor patient support system; hygiene issues; lack of an adequate medical support system in the patient’s home village; roads that are difficult to navigate compounded by landslides during the rainy season; lack of an effective means of communication once the patient returns to their home; the patient and/or family authority decides to substitute your recommendations with those of a traditional medicine doctor. Also, a particular spiritual belief system may trump Western approaches to illness.

## When to Consider Providing Equipment

There is a natural tendency among well-intentioned volunteers to donate new or used equipment. Unfortunately, if the recipients are not adequately trained in how to use the equipment, convinced of its value and/or have no system for maintaining and restocking its components, your gift will soon be relegated to the back of a closet. Occasionally, the equipment is sold for personal profit. A better choice might be modest yet durable items such as a tourniquet, small scissors, a mini-curette, a set of small osteotomes, a freer elevator, and a mini rongeur rather than a wrist arthroscopy system or screw fixation set. If a volunteer has access to substantial financial resources or can raise money through crowdsourcing, it might be wise to consider low-tech alternatives such as a used OR table in good working order. In contrast, if a volunteer has modest funds, a wise choice might be to finance the fabrication of a hand table made by a local artisan. When little or no funds are available a volunteer can often add value by focusing on strategies to upgrade sterile technique in the OR, on the wards, and in the therapy department. Also, it is very common to find that the equipment in the OR is in disarray. Categorizing, streamlining, and reviewing the potential uses of a hospital’s inventory can add benefit. One of the keys to success when tackling such issues is having a strong advocate within the department.

When a volunteer is enthusiastically engaged with their hosts, there is a tendency to make promises that may be hard to keep. These might include assurances that you will return or commitments to send equipment or facilitate the funding of a certain project. Despite good intentions, if promises are not fulfilled it can negatively affect the morale of our hosts. Volunteers are ambassadors. It is important for us to remain true to our word.

## Final Thoughts

Humanitarian work can be an extremely rewarding experience for the volunteer and those we wish to serve. It can enhance one’s ability to think creatively, adapt to unfamiliar circumstances, reinforce the value of respecting other points-of-view, broaden our cultural experience, and enhance our compassion, humility, and patience. Volunteering overseas frequently expands our medical knowledge, enriches our professional relationships, and reinforces our sense of appreciation for the support we receive in our own medical communities.

Furthermore, one should not underestimate the profound impact that working overseas can have on enhancing personality traits that can serve us well in our interpersonal relationships, physical and mental well-being, lifestyle choices, and spiritual maturation. Involving family members can help strengthen those relationships, leaving an indelible impression on your children, and providing you with an excellent opportunity to lead by example.

I have learned so much from my gracious hosts and their communities. I feel honored and humbled by their willingness to share their lives with me. It is my hope that my ongoing efforts will help me to become a better physician and more well-rounded, compassionate human being.

## Author Contributions

JK has been doing international humanitarian work for 20 years through Health Volunteers Overseas. This article is a reflection of his experience.

## Conflict of Interest Statement

The author declares that the research was conducted in the absence of any commercial or financial relationships that could be construed as a potential conflict of interest.
